# Near-Chromosome-Level Genome Assembly of the Dark Septate Endophyte *Laburnicola rhizohalophila*: A Model for Investigating Root-Fungus Symbiosis

**DOI:** 10.1093/gbe/evab026

**Published:** 2021-02-11

**Authors:** Xinghua He, Zhilin Yuan

**Affiliations:** 1 State Key Laboratory of Tree Genetics and Breeding, Chinese Academy of Forestry, Beijing, China; 2 Nanjing Forestry University, Nanjing, China; 3 Research Institute of Subtropical Forestry, Chinese Academy of Forestry, Hangzhou, China

**Keywords:** de novo genome assembly, long-read sequencing, symbiosis

## Abstract

The novel DSE *Laburnicola rhizohalophila* (Pleosporales, Ascomycota) is frequently found in the halophytic seepweed (*Suaeda salsa*). In this article, we report a near-chromosome-level hybrid assembly of this fungus using a combination of short-read Illumina data to polish assemblies generated from long-read Nanopore data. The reference genome for *L. rhizohalophila* was assembled into 26 scaffolds with a total length of 64.0 Mb and a N50 length of 3.15 Mb. Of them, 17 scaffolds approached the length of intact chromosomes, and 5 had telomeres at one end only. A total of 10,891 gene models were predicted. Intriguingly, 27.5 Mb of repeat sequences that accounted for 42.97% of the genome was identified, and long terminal repeat retrotransposons were the most frequent known transposable elements, indicating that transposable element proliferation contributes to its increased genome size. BUSCO analyses using the Fungi_odb10 data set showed that 95.0% of genes were complete. In addition, 292 carbohydrate active enzymes, 33 secondary metabolite clusters, and 84 putative effectors were identified in silico. The resulting high-quality assembly and genome features are not only an important resource for further research on understanding the mechanism of root-fungi symbiotic interactions but will also contribute to comparative analyses of genome biology and evolution within Pleosporalean species.

**Significance**

Dark septate endophytes (DSEs) represent a unique fungal group dominating plant roots, and often improve host abiotic stress tolerance. DSEs are under increasing scrutiny for their key ecological roles in terrestrial ecosystems. However, only few genomes of DSEs are currently available. In this work, the high-quality genome assembly and annotation of the novel DSE fungus *Laburnicola rhizohalophila* sp. nov. are provided. By sequencing this fungus, we could not only have a better understanding of the mechanisms underlying root-fungus symbiotic interactions, but also perform comparative analysis of genome biology and evolution between different symbiotic fungal groups.

## Introduction

Dark septate endophytes (DSEs) are a unique group of root-colonizing fungi that have recently received considerable attention, due not only to their wide host ranges but also their potential to mineralize organic matters and improve stress resistance in hosts (Mandyam and Jumpponen 2005; Knapp et al. 2012; Berthelot et al. 2016; Qin et al. 2017; Knapp et al. 2018; Vergara et al. 2018; Mateu et al. 2020). It has been increasingly appreciated that DSEs often occur simultaneously with both arbuscular mycorrhizal and ectomycorrhizal fungi and the colonization rates of these multiple fungal symbionts differed greatly in response to elevated atmosphere CO_2_ and warming, and in turn alter plant response to global change (Olsrud et al. 2009; Kivlin et al. 2013).

Despite DSEs dominating several biomes and climatic regions, the mechanisms underlying DSE–plant symbiotic relationships and their genomic evolution and adaptation are still elusive (Knapp et al. 2018). In the past few years, genome sequences of several important DSEs species, such as the *Phialocephala fortinii* s.l.–*Acephala applanata* species complex (Grünig et al. 2008; Schlegel et al. 2016), *Harpophora oryzae* (Xu et al. 2015), *Phialocephala subalpine* (Schlegel et al. 2016), *Microdochium bolleyi* (David et al. 2016), *Cadophora* sp., and *Periconia marospinosa* (Knapp et al. 2018), across a wide range of ascomycetes, are currently available.

Prior to this work, we identified a novel DSE fungus, *Laburnicola rhizohalophila* sp. nov. (Yuan et al. 2016, 2020) from roots of a halophyte seepweed (*Suaeda salsa*). Experimental resynthesis confirmed that *L. rhizohalophila* successfully colonized root tissues and showed phytobeneficial effects (Yuan et al. 2020). The availability of the *L. rhizohalophila* genome will increase our knowledge of this novel root-fungi symbiosis. To this end, we combined Nanopore long-reads and Illumina short-reads to obtain a high-quality hybrid genome assembly. Moreover, we annotated the secondary metabolite biosynthetic gene clusters, carbohydrate-active enzymes (CAZymes), and potential effectors.

## Materials and Methods

### Fungi Culture and DNA Extraction

The dried and living cultures of *L. rhizohalophila* isolate R22-1 are deposited in the Herbarium of Institute of Microbiology, Academia Sinica (HMAS 248145) and China General Microbiological Culture Collection Center (CGMCC 3.19615), respectively. The isolate was cultured in modified Melin-Norkrans liquid medium (Marx 1969) at room temperature in the dark for 1 week. The mycelium was harvested by filtration through Whatman filter paper, washed with distilled water, flash-frozen in liquid nitrogen, and ground into a powder. Genomic DNA was extracted from 2.5 g mycelia using a modified Cetyltrimethyl Ammonium Bromide protocol (Watanabe et al. 2010). DNA concentration and purity were determined using a Qubit fluorometer and Nanodrop 2000 spectrophotometer (Thermo Fisher Scientific, Carlsbad, CA). DNA integrity was assessed via 0.5% agarose gel electrophoresis. The strain collection is compliant with the Nagoya Protocol.

### Library Construction and Genome Sequencing

Whole genome sequencing was performed on the Illumina HiSeq2500 and Oxford Nanopore Technologies PromethION P24 device at BGI (Shenzhen, China). Long reads generated from the Nanopore platform were used for genome assembly, and the short but accurate reads from the Illumina platform were analyzed for genome survey and base-level correction after assembly. For the Illumina sequencing library, the insert size was 350 bp with a pair-end sequencing length of 150 bp; it was constructed using the NEBNext Ultra II DNA Library Prep Kit for Illumina (NEB). A Nanopore 20-kb insert library was prepared with 1 µg genomic DNA using a ligation sequencing kit SQK-LSK109 (Oxford Nanopore Technologies). The constructed library was quantified using a Qubit DNA HS Assay Kit in a Qubit fluorometer (Thermo Fisher Scientific, MA).

### Genomic Assembly and Assessment

NECAT (version 0.0.1_20200803, https://github.com/xiaochuanle/NECAT) was used to assemble the Nanopore long-reads data with default parameters (Chen et al. 2020). After the assembly step, each set of scaffolds was polished with Pilon (version 1.22) using Illumina cleaned reads. To improve the assembly, a second Pilon polishing step was conducted as described above (Walker et al. 2014). The bacterial contamination was identified by aligning the assembled scaffolds to the bacterial sequence database from National Center for Biotechnology Information (NCBI) using the BlastN alignment algorithm. The completeness and accuracy of the genome-assembly and gene predictions were evaluated using Benchmarking Universal Single-Copy Orthologs (BUSCO version 4.0.6) with fungus lineage-specific single-copy orthologs (Fungi_odb10) data set (Simão et al. 2015).

### Gene Prediction and Annotation

Protein-coding genes were annotated based on homology and ab initio predictions. For homology-based prediction, protein sequences of *Birnuria novae-zelandiae*, *Karstenula rhodostoma*, and *Paraconiothyrium sporulosum* were obtained from a JGI Genome Portal at the MycoCosm database (https://genome.jgi.doe.gov/programs/fungi/index.jsf). The three species were genetically close to *L. rhizohalophila*. For ab initio prediction, GeneMark-ES was performed on the repeat-masked genome using the fungal module (Ter-Hovhannisyan et al. 2008). Then, EVidenceModeler (EVM, version 1.1.1) was applied to combine all gene models that were predicted by homology and ab initio to form comprehensive and nonredundant reference gene sets (Haas et al. 2008). Public biological function databases, including the nonredundant protein sequences (NR), Kyoto Encyclopedia of Genes and Genomes (KEGG), Swissprot, and TrEMBL databases (Zhao et al. 2012), were used for functional annotation of the predicted genes using RAPSearch2 (version 2.22) (Zhao et al. 2012) applying HSSP criteria. The InterPro database (Finn et al. 2017) was used to predict protein function based on PFAM domains annotated by the InterproScan tool and having an *E*-value < 1*e*-05 (Jones et al. 2014). Gene Ontology (GO) terms were assigned to the genes using the Blast2GO pipeline.

Transposable elements (TEs) were identified using homolog-based and ab initio strategies. For ab initio predictions, RepeatModeler (version 1.0.11, http://repeatmasker.org/RepeatModeler/), RepeatScout version 1.0.5, Piler, and LTR_FINDER (http://tlife.fudan.edu.cn/tlife/ltr_finder/, Jurka et al. 2005; Xu and Wang 2007) were performed with default parameters. Tandem repeats were also predicted ab initio using Tandem Repeats Finder (TRF, version 4.0.9b). RepeatMasker (version 4.07) and the associated RepeatProteinMask were used for homologous comparisons by searching against Repbase (version 23.06, http://www.girinst.org/repbase) (Bao et al. 2015).

#### Annotation of Specific Gene Categories

Genes and gene clusters involved in secondary metabolism were predicted using antiSMASH version 4.0.2 (Blin et al. 2017). To identify the CAZyme repertoire of *L. rhizohalophila*, three tools for CAZyme annotation in dbCAN2 (http://cys.bios.niu.edu/dbCAN2) were combined: HMMER searches against the dbCAN hidden Markov model (HMM) database, DIAMOND searches against the CAZy pre-annotated CAZyme sequence database, and Hotpep searches against the conserved CAZyme short peptide database (Zhang et al. 2018). Only CAZyme domains that were predicted by at least two of the three algorithms were kept.

The secretome was predicted by screening the predicted proteins for different features using a bundle of eight different prediction tools implemented in the web-based program SECRETOOL (Cortázar et al. 2014, http://genomics.cicbiogune.es/SECRETOOL/STP_Parser.php). Then, we used EffectorP version 2.0 (http://effectorp.csiro.au/) to improve the effector prediction based on an EffectorP score (effector probability) > 0.5 (Sperschneider et al. 2016, 2018).

### Phylogeny Construction

The protein sequences for 36 species were retrieved from JGI and Ensembl Fungi, and mainly consisted of all current available genomes within Pleosporales and some DSE and ericoid mycorrhizal fungi in Helotiales. A phylogenetic tree was constructed using 761 core single-copy orthologs after filtering (< 200 amino acids). The amino acid sequences of single-copy orthologous groups were aligned using Muscle version 3.8.31. The well-aligned, conserved blocks of these alignments were extracted using Gblocks version 0.91 b with default parameters. The concatenated alignment was used to infer a phylogeny with the maximum likelihood method using RAxMLversion 8.2.12 with 1,000 bootstrap replicates. We chose the PROTGAMMAWAG model of evolution for analysis.

## Results and Discussion

### Genome Assembly and Statistics

The genome structure and locations of transposable and repetitive elements, putative effectors, CAZymes, and secondary metabolite clusters were graphed on a Circos plot (supplementary fig. S1, Supplementary Material online). A total of 8.27 Gb Nanopore long reads and 6.84 Gb Illumina short reads were generated with an estimated 129.2× average depth of sequencing coverage.

After assembly and polishing, we obtained a final genome without gaps of 64.0 Mb, with 26 scaffolds, an N50 length of 3.15 Mb, and an overall GC content of 44.4% (table 1). The maximum scaffold size exceeded 4.9 Mb (table 1). The assembly size is the second largest of the published genomes in the Pleosporales (supplementary table S1, Supplementary Material online). Of the 26 scaffolds, 17 scaffolds had a characteristic telomere sequence (5′-TTAGGG-3′) at both ends, indicating that the 17 scaffolds approach the length of intact chromosomes (Aksenova and Mirkin 2019), and 5 scaffolds contained telomeric repeat sequences on the 5′ or 3′ end. Homology analysis of assembled scaffolds supports an absence of bacterial contamination in our genome assembly.

**Table 1:** Summary Assembly and Annotation Statistics for Nanopore Long-Read Sequencing of *Laburnicola rhizohalophila* R22-1

Assembly Statistics	R22-1
Genome size (bp)	64,007,018
Total sequenced bases	∼8.27 Gb
Coverage	129.20
Number of scaffolds	26
Largest scaffold size	4,903,573
N50 (bp)	3,149,867
G+C content (%)	44.40
Complete chromosomes	17
Number of protein coding genes	10,891
Predicted secreted proteins	376
Predicted small secreted proteins (SSPs)	181
Predicted effectors	84

BUSCO evaluation revealed that the genome completeness reached 98.02% (a total of 758 BUSCO groups searched) (Simão et al. 2015), and 1 and 14 BUSCO orthologs were fragmented (0.13%) and missing (1.85%), respectively. These results suggest good integrity of the assembled genome (table 1).

### Genomic Repeats

Multivariate repeated DNA sequences may account for variation in genome size (Biemont, 2008). In all, ∼27.5 Mb repeat sequences that accounted for 42.97% of the genome were identified by four repeat annotation processes (supplementary table S2, Supplementary Material online). The most abundant of the transposable and repetitive element types were Class I, long terminal repeats (LTRs) with 16.30 Mb (25.46%), Class II DNA transposons (DNA), with 8.03 Mb (12.55%) (supplementary table S3, Supplementary Material online). Compared with other Pleosporalean fungi included in this study, *L. rhizohalophila* had a larger genome (Mohanta and Bae 2015; Knapp et al. 2018) (supplementary table S1, Supplementary Material online). As previously reported, TEs are positively correlated with genome size (Kidwell 2002; Hua-Van et al. 2005; Biscotti et al. 2015). Our data also suggest that TE content in 19 Pleosporalean genomes was positively correlated to genome assembly size (Pearson's correlation = 0.631, *P*-value = 3.0*e*-05) (supplementary fig. S2, Supplementary Material online). Therefore, the larger genome of *L. rhizohalophila* is mainly driven by TE proliferation. Many plant pathogenic fungi have an increased TE content, particularly (hemi) biotrophic and symbiotic fungi (Raffaele and Kamoun 2012; Peter et al. 2016).

### Protein-Coding Gene Prediction and Functional Annotation

We ultimately generated a gene set of 10,981 protein coding genes with an average length of 1,377 bp (table 1). Among them, 10,796 (99.13%) could be annotated with at least one database (InterProScan, Gene Ontology, Kyoto Encyclopedia of Genes and Genomes, and SwissProt). Specifically, 10,657 proteins (97.85%) had significant InterPro hits, and 9,741 proteins (89.44%) had GO annotations. BUSCO assessment on gene annotation showed that the annotation completeness reached 95.0%, and only 14 (1.8%) and 24 (3.2%) BUSCO orthologs were fragmented and missing, respectively.

### Secondary Metabolite Biosynthetic Clusters

**Fig. 1:**
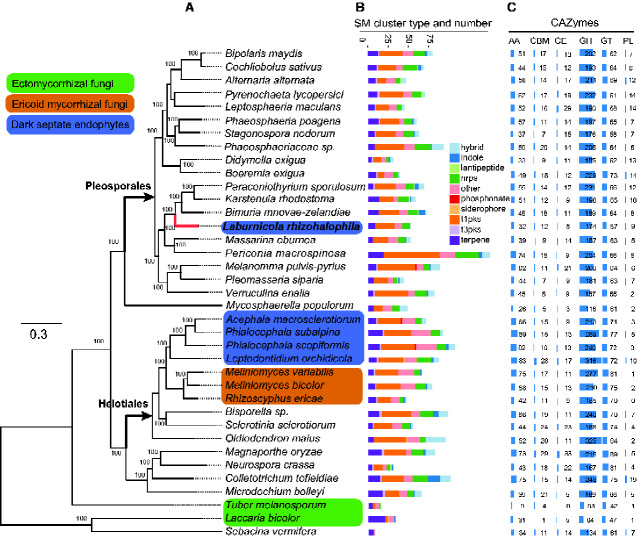
Maximum likelihood species phylogeny of the 36 fungal species used in this study (*A*). Two basidiomycetes, *Laccaria bicolor* and *Sebacina vermifera*, were used as outgroups. Three groups of mutualistic root fungi including dark septate endophytes, ericoid mycorrhizal fungi, and ectomycorrhizal fungi are indicated in blue, brown, and green, respectively. Bootstrap values of 100% are indicated above the nodes; the arrow indicates the base of the Pleosporales and Helotiales. The parameters used in the ML include the GTRGAMMA model of evolution and 1,000 bootstrap replicates for branch support estimation. (*B*) Number of gene clusters encoded for secondary metabolism enzymes predicted for each of the 46 fungal species using antiSMASH v4.0.2, shown as a bar chart. T1PKS, type I polyketide synthases; T3PKS, type III PKSs; NRPS, nonribosomal peptide synthases; hybrid, NRPS/PKS hybrid. (*C*) Comparison of the relative number of genes per funtional group of CAZymes. GH, glycoside hydrolases; GT, glycosyl transferases; PL, polysaccharide lyases; CE, carbohydrate esterases; AA, auxiliary activity enzymes; CBM, carbohydrate-binding modules.

We detected a total of 33 secondary metabolite biosynthetic gene clusters in *L. rhizohalophila*. Of them, 12 belonged to the type I polyketide synthases (PKS) group, 8 to the nonribosomal peptide synthases (NRPS), 5 to the terpene synthase group, and the remaining clusters are unknown (fig. 1). The gene clusters were not equally distributed over the chromosomes (or scaffolds), and were often located subtelomerically (supplementary fig. S1, Supplementary Material online).

### CAZyme Repertoire

The fungi produced a diverse array of CAZymes for nutrition and plant infection. Overall, the genome of *L. rhizohalophila* contained only 292 genes encoding putative CAZymes, considerably lower than the average in Pleosporales and Helotiales, only slightly larger than *Laccaria bicolor* (149), *Neurospora crassa* (213), and *Tuber melanosporum* (109) (fig. 1). Plant cell wall degrading enzymes (PCWDEs) are mainly distributed in the CE, GH, and PL classes (Chang et al. 2016). In the case of *L. rhizohalophila*, the number of PCWDE-related genes (174 GHs, 9 PLs, and 8 CEs) was also lower than in most other fungal members (fig. 1). This scenario is consistent with some ectomycorrhizal fungi, in which their genomic idiosyncrasies are often accompanied by a restricted set of PCWDEs (Peter et al. 2016). Low-diversity PCWDE genes are often imprinted in the genomes of biotrophs (both symbiotic and pathogenic) to avoid triggering plant defense mechanisms (Spanu 2012), thus facilitating adaptation to their biotrophic lifestyles (Veneault-Fourrey and Martin 2011). Our data further support a convergent decrease in plant cell wall degrading capacity of ectomycorrhizal and DSE fungi (Kohler et al. 2015).

### Secreted Proteins and Effectors

With the SECRETOOL pipeline, 376 proteins were predicted to be secreted, which is 3.42% of the whole predicted proteome of *L. rhizohalophila*. Within the secretome, small secreted proteins (SSPs) with sequence lengths of <300 amino acids have been widely studied for their role in fungus–plant interactions, and 181 SSPs were predicted in silico. A few of the SSPs have been found to act as effectors that manipulate plant immune responses in plants. We further divided the SSPs into effector based on certain sequence characteristics, such as size and cysteine content, and ultimately predicted 84 potential effector candidates with probabilities ranging from 0.56 to 0.97 (table 1).

### Phylogenomics of *L. rhizohalophila*

The amino acid sequences of 761 single-copy orthologous groups were used to construct a genome-based maximum-likelihood (ML) tree in which *L. bicolor* and *Sebacina vermifera*, two basidiomycetous species, were treated as the outgroups. The topology of the phylogenetic tree was strongly supported by 100% bootstrap values on most branches (fig. 1). According to this phylogram, *Bimuria novae-zelandiae* was the most closely related species to *L. rhizohalophila*, followed by *K. rhodostoma*, and *P. sporulosum*. All of them belonged to the Didymosphaeriaceae family (Pleosporales, Dothideomycetes).

## Supplementary Material

Supplementary data are available at *Genome Biology and Evolution* online.

## Supplementary Material

evab026_Supplementary_Data
