# Supplementary material for: Near-Chromosome-Level Genome Assembly of the Dark Septate Endophyte Laburnicola rhizohalophila: A Model for Investigating Root-Fungus Symbiosis
Source: Genome Biol Evol. 2021 Feb 11;13(3):evab026. doi: 10.1093/gbe/evab026 (PMC7936028; doi:10.1093/gbe/evab026)
Supplement: evab026_Supplementary_Data [file evab026_supplementary_data.zip › Supplementary figure legends.docx]

**Figure S1** Circos plot of key genomic features of the *Laburnicola rhizohalophila* R22-1 reference genome. The 21 largest scaffolds; the remaining 5 scaffolds are not shown due to their short length of <200 Kb. Tracks labeled from outside: (A) Distribution of annotated genes (gray) and telomeres (blue); (B) Locations of predicted effector genes (red), CAZyme genes (green), and predicted secondary metabolite clusters (purple); (C) gene density with 20 Kb moving average window; (D) Percent GC content with a 50 Kb moving average window; (E) Repeat type: Copia (green), DNA transposons (blue), Gypsy (red) , others (yellow), simple repeat (pink); (F) TE and repetitive DNA density with a 20 Kb moving average window.

**Figure S2** Relationship between TE coverage and [genome size](https://www.sciencedirect.com/topics/biochemistry-genetics-and-molecular-biology/genome-size). Dots: individual measurements; black line: linear regression; blue area: confidence interval.
