# Supplementary material for: Near-Chromosome-Level Genome Assembly of the Dark Septate Endophyte Laburnicola rhizohalophila: A Model for Investigating Root-Fungus Symbiosis
Source: Genome Biol Evol. 2021 Feb 11;13(3):evab026. doi: 10.1093/gbe/evab026 (PMC7936028; doi:10.1093/gbe/evab026)
Supplement: evab026_Supplementary_Data [file evab026_supplementary_data.zip › Table S1 Genome assembly statistics for the nineteen species in Pleosporales.doc]

**Table S1 Genome assembly and annotation statistics for the nineteen species in Pleosporales**

| Species | Assembly (Mb) | Scaffolds | N50 (Mb) | Gap (%) | TEs (%) | Genes | BUSCO | Sequencing | Annotation |
| --- | --- | --- | --- | --- | --- | --- | --- | --- | --- |
| *Alternaria alternata* | 33.44 | 26 | 3.09 | 0 | 1.18 | 13,086 | 99.3 | PacBio | JGI |
| *Bimuria novae-zelandiae* | 78.18 | 321 | 0.71 | 1.8 | 47.42 | 16,681 | 99.6 | Illumina | JGI |
| *Bipolaris maydis* | 36.46 | 68 | 1.84 | 0.4 | 9.88 | 13,336 | 99.5 | Sanger | JGI |
| *Boeremia exigua* | 35.32 | 26 | 2.53 | 0 | 4.51 | 12,871 | 99.3 | PacBio | JGI |
| *Cochliobolus sativus* | 34.42 | 157 | 1.79 | 3.5 | 7.72 | 12,250 | 99.3 | Sanger/Illumina/454 | JGI |
| *Didymella exigua* | 34.39 | 176 | 0.84 | 2.1 | 14.33 | 12,394 | 98.6 | Solexa | JGI |
| *Karstenula rhodostoma* | 45.07 | 139 | 1.93 | 1 | 1.23 | 15,043 | 99.3 | Illumina | JGI |
| *Laburnicola rhizohalophila* | 64.01 | 26 | 3.15 | 0 | 40.13 | 10,891 | 95.0 | Nanopore/Illumina | JGI |
| *Leptosphaeria maculans* | 44.89 | 41 | 1.77 | 2.5 | 39.93 | 12,469 | 95.7 | Sanger | JGI |
| *Massarina eburnea* | 38.24 | 233 | 1.57 | 3.6 | 13.40 | 12,935 | 99.0 | Illumina | JGI |
| *Melanomma pulvis-pyrius* | 42.09 | 1,325 | 0.06 | 0 | 3.65 | 15,881 | 99.4 | Illumina | JGI |
| *Paraconiothyrium sporulosum* | 38.46 | 61 | 3.13 | 1 | 2.47 | 14,745 | 99.5 | Illumina | JGI |
| *Periconia macrospinosa* | 54.99 | 1,566 | 0.14 | 1.8 | 10.80 | 18,750 | 98.5 | Illumina | JGI |
| *Phaeosphaeriaceae* | 56.37 | 591 | 0.33 | 0 | 28.05 | 15,864 | 97.7 | PacBio/Illumina | JGI |
| *Phaeosphaeria poagena* | 34.08 | 26 | 1.63 | 0 | 2.35 | 12,804 | 98.9 | PacBio | JGI |
| *Pleomassaria siparia* | 43.18 | 193 | 1.89 | 1.3 | 19.88 | 13,486 | 99.3 | Illumina | JGI |
| *Pyrenochaeta lycopersici* | 40.27 | 241 | 0.54 | 0 | 4.68 | 14,311 | 97.5 | PacBio | JGI |
| *Stagonospora nodorum* | 37.21 | 108 | 1.05 | 0.4 | 5.97 | 12,380 | 93.9 | Sanger | JGI |
| *Verruculina enalia* | 61.21 | 611 | 0.25 | 5.3 | 31.97 | 13,748 | 99.4 | Illumina | JGI |

BUSCO: benchmarking universal single-copy orthologues. Note that this BUSCO is an assessment of the completeness for gene predictions.
