# Supplementary material for: Near-Chromosome-Level Genome Assembly of the Dark Septate Endophyte Laburnicola rhizohalophila: A Model for Investigating Root-Fungus Symbiosis
Source: Genome Biol Evol. 2021 Feb 11;13(3):evab026. doi: 10.1093/gbe/evab026 (PMC7936028; doi:10.1093/gbe/evab026)
Supplement: evab026_Supplementary_Data [file evab026_supplementary_data.zip › Table S2 Summary statistics of repeat annotation in the genome of L.doc]

**Table S2** Summary statistics of repeat annotation in the genome of *L. rhizohalophila* R22-1 using TRF, RepeatMasker, and RepeatProteinMask. Note that the total content was merged and redundancy was eliminated by each method.

| Type | Repeat size (bp) | % of genome |
| --- | --- | --- |
| Trf (Tandem Repeat Finder) | 1,157,012 | 1.81 |
| RepeatMasker | 5,796,016 | 9.06 |
| RepeatProteinmask | 8,122,413 | 12.69 |
| *Ab initio* | 26,745,530 | 41.79 |
| Total | 27,502,772 | 42.97 |
