# Supplementary material for: Near-Chromosome-Level Genome Assembly of the Dark Septate Endophyte Laburnicola rhizohalophila: A Model for Investigating Root-Fungus Symbiosis
Source: Genome Biol Evol. 2021 Feb 11;13(3):evab026. doi: 10.1093/gbe/evab026 (PMC7936028; doi:10.1093/gbe/evab026)
Supplement: evab026_Supplementary_Data [file evab026_supplementary_data.zip › Table S3 Summary statistics of repeat annotation in L.doc]

**Table S3** Summary statistics of repeat annotation in *L. rhizohalophila* strain R22-1.

| Type | Repbase+*Ab initio* | | TE proteins | | Combined TEs | |
| --- | --- | --- | --- | --- | --- | --- |
|  | Length (Mb) | % in genome | Length  (Mb) | % in genome | Length  (Mb) | % in genome |
| DNA transposons | 7.55 | 11.79 | 3.96 | 6.19 | 8.03 | 12.55 |
| LINE | 0.44 | 0.68 | 0.27 | 0.42 | 0.59 | 0.93 |
| SINE | 0.00 | 0.00 | 0.00 | 0.00 | 0.00 | 0.00 |
| LTR | 16.16 | 25.25 | 3.89 | 6.07 | 16.30 | 25.46 |
| Unknown | 4.46 | 6.97 | 0.00 | 0.00 | 4.46 | 6.97 |
| Total | 25.11 | 40.17 | 8.12 | 12.69 | 25.77 | 40.25 |

Note: Repbase + De novo: RepeatMasker results based on Repbase, RepeatModeler, RepeatScout, Piler, and LTR_FINDER; TE proteins: RepeatProteinMask results based on Repbase; Combined TEs: combined results of De novo + Repbase and TE proteins. LINE: long interspersed nuclear element; SINE: short interspersed nuclear element; TE: transposable element
